# Supplementary figures and images for: Investigating the regulatory role of HvANT2 in anthocyanin biosynthesis through protein–motif interaction in Qingke
Source: PeerJ. 2024 Jul 10;12:e17736. doi: 10.7717/peerj.17736 (PMC11246018; doi:10.7717/peerj.17736)

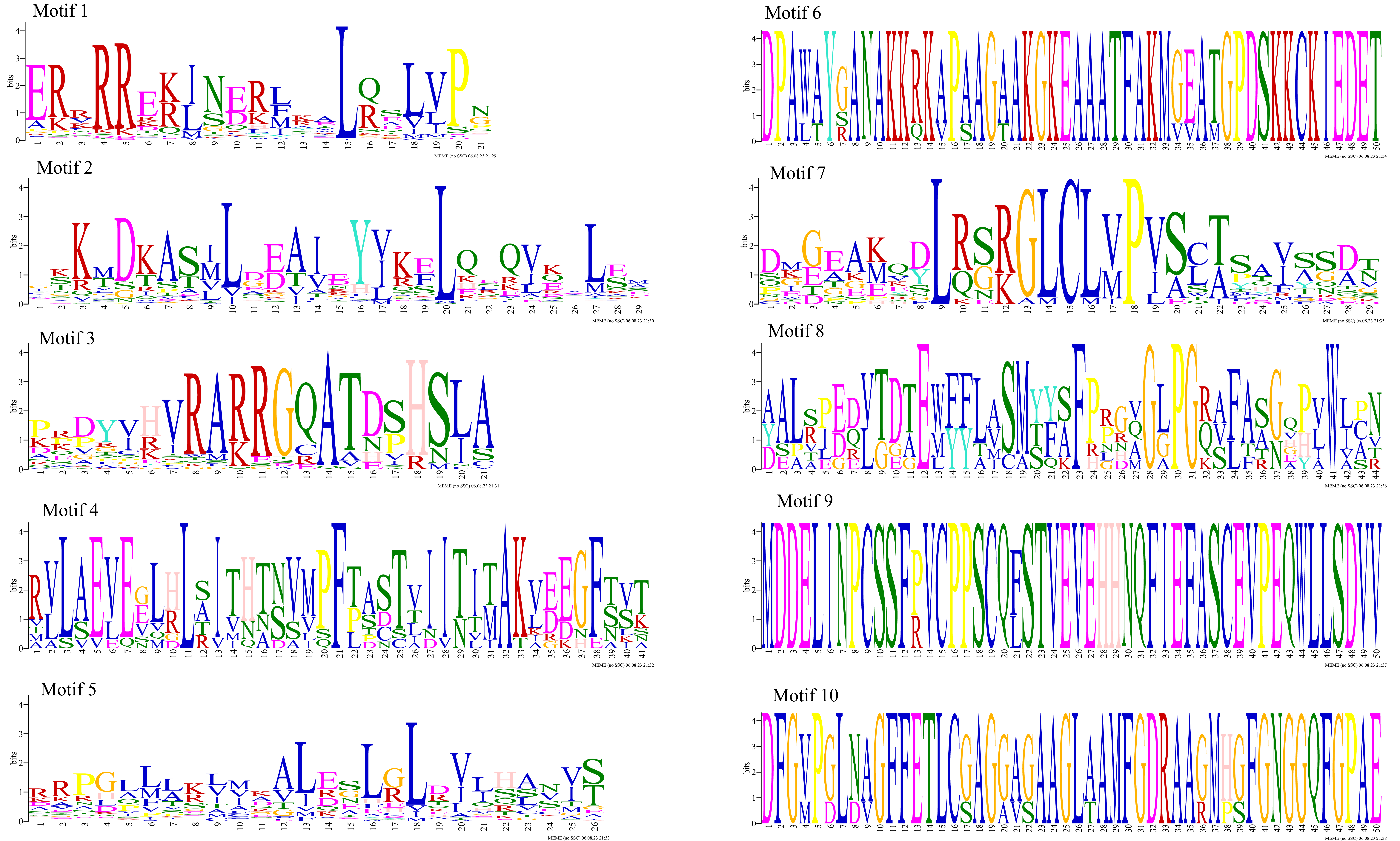

Supplement: Supplemental Information 1 [file peerj-12-17736-s001.png]

SD-TLH+50 mM 3AT CHI


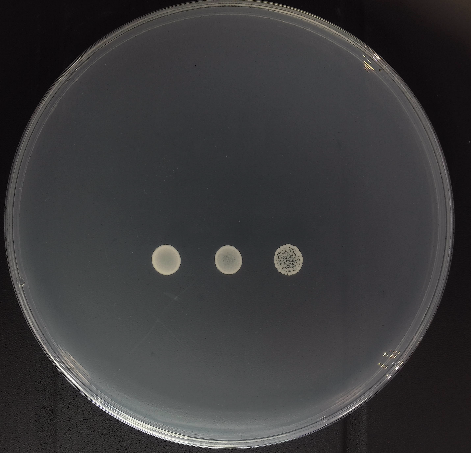

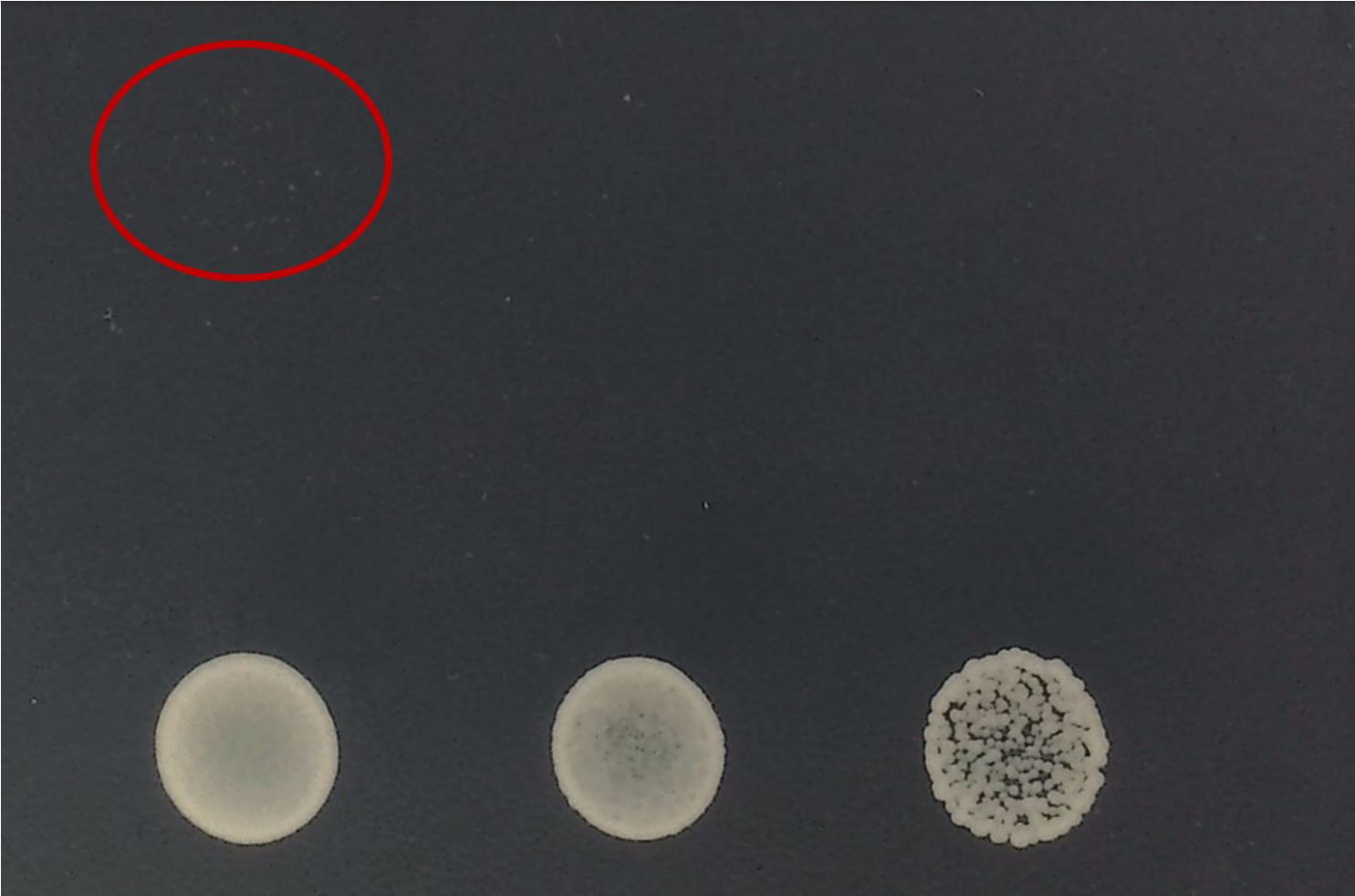


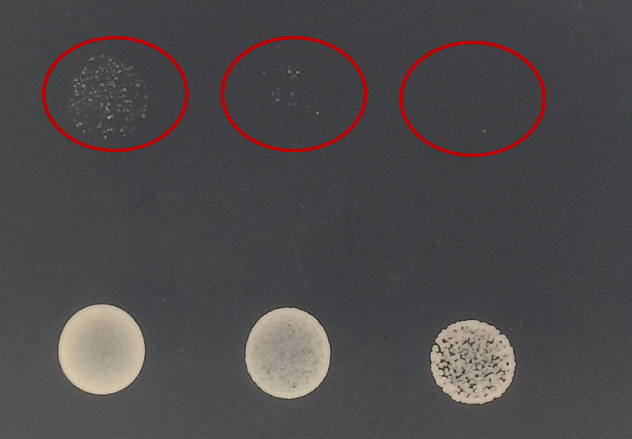

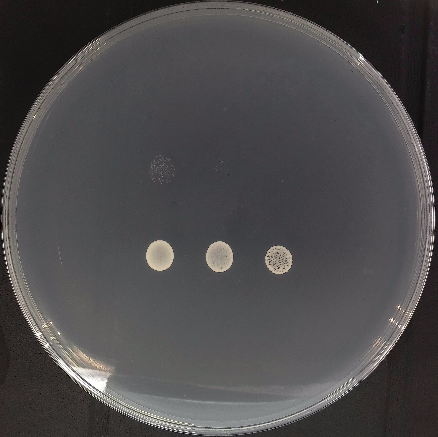
SD-TLH+50 mM 3AT GT

Supplement: Supplemental Information 6 [file peerj-12-17736-s006.docx]
