# Supplementary material for: Investigating the regulatory role of HvANT2 in anthocyanin biosynthesis through protein–motif interaction in Qingke
Source: PeerJ. 2024 Jul 10;12:e17736. doi: 10.7717/peerj.17736 (PMC11246018; doi:10.7717/peerj.17736)
Supplement: Supplemental Information 2 [file peerj-12-17736-s002.docx]

| gene name | Promoter sequences of genes |
| --- | --- |
| *CHI* | ATTTTGTGTGAATTAATTTCTCAAACTCCGACTATCCATGGCGCTGAAGATAAGTCGACATGATTTAGGATCCGAAAAGAGTTTTTGTTATTGTTATTATTATCAACAAAGTCTGTATATATATAGTACACTTTTTTGCTGCGAATATCAATTGCAATAAGGATTGCACCATCTGGTAAGCCTGGGCGCCGTTGAAAGGCATACAATTTCTTTGGTTCATCGCTAGACATATTTTTTTTCCTTTTCCTAGTTTTTTTTCAAGCGTAGGAGGATAATCACAAAAGACCGTGGATTCTTTTTTGGGAATCGTTTTCATACGGCGCTCCGGCCAAACCGACGCGCCGATCGCTTCCTTACTCGTGCGGGCCATGCAACATTTTTTCGTAGACGCGCGCTTCGTTGGTCAGTGGATGCAACATTTTTCGTCTAATTGTTGCAACCAGCGTCATATTTGCTGCAAGCGTTTTGTTAATACTTTTGTCCAGTAAAAAAACTGCATATACGTTTGGTTGCAACTCCGGTTCGTCGGATTTTTCGTTACAATCGATGTTTTTTTACTTTTGCTACAACCGTGTTAATTTTTGCTACAACCGGCATCATTTTTTGTTGCAACTGTTTACTAAAAAAGTTGCATACACGTTCATGTAGATATTTATTGCAACCGCGTTTGACTTTTGCTACCACGCACCATCAACTTTGTTTTTTTGCTACGATCACGTAGGTATTCTTTTTGCTACAAATTTTATTTTTTGTTGCAACCGTTGAAAAATTGCTGCATCACGGTGAATTTTGCTGCATCGGGAGAAAAAATGTTGCATGGAGATCCAACGATGCGGACGCGCGGAAATTGATGGATCATGCGGCTCGCGCGTGGTCGGCCGAAAGTCGCGCCGGCGCAGAGCACTGCCCTTCTTTTTTCTATCAATGGGAGCTTTCCCATCCAGGATTTATAGATATCCCTCTTAGCTCTTAGTACAAGGTGTTTATCTGACATGTTTGAATTTCATAGGGATATATGCATTGGAGGACGTATCTGGTGCACAGGAACAGTTGATGCTACCTGTGCACGGGACCCAGTTGCATATTAAAAAATGTTGAAAAAAATAAAAAAATAAAACCCATTAACACACCATCATTCGACGTTGGGTTCCTTTCCTTTACCAGACCGGCAGACCCTGCTGCGGCCACCACGAGTGTGTGTGATCCGGACCCGTACGTGGGCTGAATGGTAGCTTGACCCCGCCGCAACACCTGCCGCGCGGCCACGTCCTCTCTTTCCCCCCACAACGTGCCTCCGCCAACTACCACTATCCACGCCCCCAGATACGCCCACAAACTACATGTTTGCCACGCCCCCCTGCTCCAGCCGCAGATCACGGGCGTGGCAAAACCGCAATTACGGGCAGAGTCGACGGGCATTTACCTAGCAGTTGGGGCGTCATATACCCGAGGGCACCACCACCCCAGCTAACCATCCCGTGCGCCCACGCCTCGCTAGATCGCACCCACCTACCCCTGCCCCCATCGTCTGCTCGCTACGTGCCATCCGTCCGTCGATACACATACC |
| *F3'H* | CCACCCACCAAACCAAGTCCAACTCGGTTTGGGGGGGGGAGCCTTCCCCCCTTGGACTCGGCCGACCCCCTTGGGGCTCCTTGAGCCCCAAGGCAAGGTCCCCTCCCTCCCACCTATATATACGGAGGTATTGGGGCTGATTTGAGACGACTTTTCCACGGCAGCCCGGCCACATACCTCCACGGTTTTTCCTCTAGATCGTGTTTCTGCGGAGCTCAGGCGGAGCCCTGCTGAGACAAGGTCATCACCAACCTCCGGAGCGCCGTCACGCTGCCGGAGAACTCTTCTACCTCTCCGTCTCTCTTGCTGGATCAAGAAGGCCGAGATCATCGTCGAGCTGTACGTGTGCTGAACGCGGAGGTGCCGTCCGTTCGGTACTAGATCGTGGGACTGATTGCGGGATTGTTCGCGGGGTGGATCGAGGGACGTGAGGACGTTCCACTACATCAACCGCGTTCACTAACGCTTCTGCTGTACGATCTACAAGGGTACGTAGATCACTCATCCCCTCTCGTAGATGGACATCACCATGATAGGTCTTCGTGCGCGTAGGAAAATTTTTGTTTCCCATGCGACGTTCCCCAACACTTGTATCACAAGTGATGCATATATATCTCTTCTTTTCAGTACATGTACTTGTAACGATATCCATTCTTGCGACACGACGAGATGCGCTTCTATCCCTGATGAGGCCTTCGTGCCAAATTCAGGATAGGGTCACATCTTGAGCGTGACACTCTGTCCTGGTATCACAAGTCATGCATAACGGTTTACTCGCTACTATGCGGTACCGCCTTGCCATTGTTGCTTCTGTAGAGCTTGTGTCTATTTTATCTTTGTTGCAATGTTGCCACGAACGACGCGCCCGATGACAGGTTCCTCACCCACGGCTTCTTCTCCTCAAGGTGCGCCTACTCGTTTCTCTCCTCCGACCACGAGATGGATCTCAATGATGGGTACATCTGGAGCTCAAAGGCACCTATAAAGGTCAGAATCTACGGTTGGCTTCACTGCCGTGACAGGTTGAGCACGATGGCGAACTTACATCATAAGACCATTACCTCTTATTCGGATTGTCCGCGGTGCGCTCTCACTCATGAGGACGCATTACACATCTCCATCCTTTGCCCATACGCGGTTCAGGTATGGGCCCTCCTCACTCCATCGACCGCCTTTGGGAGACTACGACACCAGTCGGCCTCGACATCAACATTTGGCGTACTGTTGCCCTCGTCATCCTCTGGAAGCCGTGGGACTTCAGGAATGTTCGTGTCTTTCAAAACGAGCTACGCACACCCCTAGTTACTCTTCGATCGGAACCTTGTTTCGGACTTTACGTTTTGGGTCTTAAGATTGAAGGACCTACTTAGTAGGAAGGCTATCATATATTGGCGCCTATACCTCTCCTCTCGTTTTAATCTATGATGTAACCTGTCCTCGGACCTTTGAGTAATATATTCGGGTGAGGAACCTTTTCCTTCCCGTGATTGTTTAAAAAAAAAAGTAAGTGCCGCTTGCGGAAGAAAGCTACACTGAACGTGCGAGTTTGTGCCACCGTGAGTAGCTTACTCATCGCTCACTCACTCAAACGTGGTAGTTTTTTGTGCCGTGAACCGTGAGTAGTTAACATATGTGGCTGCTGAGTGAGTGATTGGAAAGGTGAGTCGTTTGTCTTGTTTCCTAGGACGGTGGACCGTGGCAAGTGCAGGTTCTTCCTTTCTCTCTTTCTTTCTTTCTTTCTTTGTCAATATATAGCTAGCTCAAAAGCCATTGGAAAGAAAGCTCAGTTTTTTGTCTTGTTTCCTTGCTTGCCACTCGTACAAATACAAATACAACTACTGAGGTGAGGTGAGGTGCCAAGTGCCATTCTTCCTCCTCCTCCGGCAGGGGCAGGGGTTAGGTAGGAGGACCGGTGGATCCGGCGATTTAGGTGGAGCACGCGGCGACGACCTCCCATTTCCTTATATATATGCATGCATGGAATGGAGCCTAACCTAACAAACACTGCAGTGCACTCTC |
| *GT* | ATCAACTGAGGGTACTCCCTTCGGTCCTTTTTACTCCGCGTATTAGATTTTGGTCAAGTCAAACTTTACAAAGTTTGATCAAATTTATATTAAAAAATGTCAATATACAATATCAAATGTATATACTATGAAATTACATTTCATAAAGAATCTAACAATATTGATTTGGCATGATAAATGTTGATAATTTTTTCTATAAATTTGATAAAGTAGAGATGTTTTGACTTCGGACAAAAAACTTATATGCAAACTAAAAAGGGCCGGAAGAAGTATCATTATACTTTTAACCGTCCAAACAATCGATCCAACCATATTGGTTCACGTATTAGATCATAAAACGTGTGTGGATCAAGGCACCATGCACTTATGTACTATGTGGATCAACAACACGAGCACGCGCGGCCACGCCAGCGAACTAAAAACATGGTTGTTGATTTTTTAGGTGTAATATAAGGTCGTTTTGAGATCGAGAGGGAGCATGCCAACAACACTGCACGCGCTTGCCCCAGCTAACTAATATAAGGCCTTCACTAATTTGTCCTCACGTAGGTCAGCTACATCACGCAGGCCATCGTCATAGCAATCAATCATGTGTTGATTGCACGGATGGGTGCTTCCATGAATGGGACGGTGGTGATTGCGGCCGGAAGCTCATTAGCTTCATGTCAAAATTTAACCTTTTTGTTGTCCATGTGTATTTAAGTCATTAAATGCATTCTAGATATTTTGTGAACTGAAGTACACAAAAAAATTAGCAAAATTGGGTTGAACAATCCTATATACATACTAATACACTAAGCTTCCTAGTTTTATGGGGCTTACAATCAATTGAGGTCTTCAAATTGAAATTGAGTCACTTGATTTTCATGTCAGCAGTTTCTCAATAGCTCTCTCATTCTATTCTTTTAAGCTTCTTAATTTTAGAGGCCTCAAAATAAATTGAGAACTATAATTTGAAATTGGGTCAGTCAATTTTAGAGGGCCCACAATCAATTGAGGTCTTCAAATTATAACTGGATTATTTAATAACAATTTCTCAAGAAACGCTCTCATTCTATTCTTGTAAGCTCCCTGATTTTAGAGACCCCACAAAAAATTTAAAAATAATTTTCTTTCACCATTCAACTTGTTTGTATGTAGTTACTATAAACGTACATGCTAGATCTATAATTTGAAAATAAATTCAAGTCATTTTAACCTTAATTGTATTATAGATCAACAGAAGAAAACTTAGGGTCAGTGTTAAGGTACTATAGCTTCTCTAACCTCTCTTGCGTATGTTAGAGAATTTGGACATGGCATTGTTGGCAAGGGGCAAACCACTACATTCACCATTCTCAGCTGTATATAATACGCACAAGAGCGGCTGCTGGATTAGAATTAGATATACACGACTACGTGTGAGACGAGAGAGCACTTGACCAAAAGCTTCTGTAGCTGCACGCGGTAAGTACG |
