# Supplementary material for: Investigating the regulatory role of HvANT2 in anthocyanin biosynthesis through protein–motif interaction in Qingke
Source: PeerJ. 2024 Jul 10;12:e17736. doi: 10.7717/peerj.17736 (PMC11246018; doi:10.7717/peerj.17736)
Supplement: Supplemental Information 3 [file peerj-12-17736-s003.docx]

| **Gene ID** | **Gene name** | **Gene ID** | **Gene name** |
| --- | --- | --- | --- |
| *HORVU.MOREX.r3.3HG0244970* | *HvbHLH1* | *HORVU.MOREX.r3.1HG0053170* | *HvbHLH82* |
| *HORVU.MOREX.r3.1HG0009860* | *HvbHLH2* | *HORVU.MOREX.r3.3HG0321590* | *HvbHLH83* |
| *HORVU.MOREX.r3.4HG0394410* | *HvbHLH3* | *HORVU.MOREX.r3.4HG0390530* | *HvbHLH84* |
| *HORVU.MOREX.r3.7HG0713430* | *HvbHLH4* | *HORVU.MOREX.r3.7HG0716830* | *HvbHLH85* |
| *HORVU.MOREX.r3.3HG0294110* | *HvbHLH5* | *HORVU.MOREX.r3.5HG0497150* | *HvbHLH86* |
| *HORVU.MOREX.r3.4HG0407160* | *HvbHLH6* | *HORVU.MOREX.r3.5HG0524270* | *HvbHLH87* |
| *HORVU.MOREX.r3.3HG0283700* | *HvbHLH7* | *HORVU.MOREX.r3.6HG0607570* | *HvbHLH88* |
| *HORVU.MOREX.r3.5HG0486320* | *HvbHLH8* | *HORVU.MOREX.r3.2HG0161630* | *HvbHLH89* |
| *HORVU.MOREX.r3.4HG0337970* | *HvbHLH9* | *HORVU.MOREX.r3.5HG0484090* | *HvbHLH90* |
| *HORVU.MOREX.r3.7HG0724770* | *HvbHLH10* | *HORVU.MOREX.r3.7HG0750020* | *HvbHLH91* |
| *HORVU.MOREX.r3.3HG0267290* | *HvbHLH11* | *HORVU.MOREX.r3.2HG0110130* | *HvbHLH92* |
| *HORVU.MOREX.r3.4HG0338770* | *HvbHLH12* | *HORVU.MOREX.r3.4HG0409010* | *HvbHLH93* |
| *HORVU.MOREX.r3.2HG0166570* | *HvbHLH13* | *HORVU.MOREX.r3.7HG0672700* | *HvbHLH94* |
| *HORVU.MOREX.r3.6HG0624240* | *HvbHLH14* | *HORVU.MOREX.r3.7HG0748480* | *HvbHLH95* |
| *HORVU.MOREX.r3.5HG0493040* | *HvbHLH15* | *HORVU.MOREX.r3.7HG0682660* | *HvbHLH96* |
| *HORVU.MOREX.r3.7HG0679990* | *HvbHLH16* | *HORVU.MOREX.r3.6HG0615060* | *HvbHLH97* |
| *HORVU.MOREX.r3.5HG0508700* | *HvbHLH17* | *HORVU.MOREX.r3.7HG0706460* | *HvbHLH98* |
| *HORVU.MOREX.r3.5HG0438460* | *HvbHLH18* | *HORVU.MOREX.r3.5HG0515900* | *HvbHLH99* |
| *HORVU.MOREX.r3.7HG0679760* | *HvbHLH19* | *HORVU.MOREX.r3.2HG0195460* | *HvbHLH100* |
| *HORVU.MOREX.r3.4HG0350240* | *HvbHLH20* | *HORVU.MOREX.r3.3HG0252710* | *HvbHLH101* |
| *HORVU.MOREX.r3.4HG0392660* | *HvbHLH21* | *HORVU.MOREX.r3.7HG0727850* | *HvbHLH102* |
| *HORVU.MOREX.r3.4HG0387050* | *HvbHLH22* | *HORVU.MOREX.r3.5HG0492770* | *HvbHLH103* |
| *HORVU.MOREX.r3.5HG0519660* | *HvbHLH23* | *HORVU.MOREX.r3.1HG0032650* | *HvbHLH104* |
| *HORVU.MOREX.r3.3HG0309530* | *HvbHLH24* | *HORVU.MOREX.r3.2HG0153790* | *HvbHLH105* |
| *HORVU.MOREX.r3.7HG0660920* | *HvbHLH25* | *HORVU.MOREX.r3.1HG0056640* | *HvbHLH106* |
| *HORVU.MOREX.r3.6HG0592420* | *HvbHLH26* | *HORVU.MOREX.r3.2HG0138530* | *HvbHLH107* |
| *HORVU.MOREX.r3.4HG0398470* | *HvbHLH27* | *HORVU.MOREX.r3.2HG0158950* | *HvbHLH108* |
| *HORVU.MOREX.r3.4HG0415230* | *HvbHLH28* | *HORVU.MOREX.r3.7HG0708530* | *HvbHLH109* |
| *HORVU.MOREX.r3.7HG0731430* | *HvbHLH29* | *HORVU.MOREX.r3.6HG0608480* | *HvbHLH110* |
| *HORVU.MOREX.r3.2HG0122430* | *HvbHLH30* | *HORVU.MOREX.r3.6HG0603090* | *HvbHLH111* |
| *HORVU.MOREX.r3.5HG0458030* | *HvbHLH31* | *HORVU.MOREX.r3.6HG0580040* | *HvbHLH112* |
| *HORVU.MOREX.r3.7HG0684680* | *HvbHLH32* | *HORVU.MOREX.r3.7HG0654120* | *HvbHLH113* |
| *HORVU.MOREX.r3.5HG0490730* | *HvbHLH33* | *HORVU.MOREX.r3.7HG0652460* | *HvbHLH114* |
| *HORVU.MOREX.r3.5HG0512260* | *HvbHLH34* | *HORVU.MOREX.r3.7HG0750050* | *HvbHLH115* |
| *HORVU.MOREX.r3.7HG0652470* | *HvbHLH35* | *HORVU.MOREX.r3.3HG0224000* | *HvbHLH116* |
| *HORVU.MOREX.r3.7HG0702010* | *HvbHLH36* | *HORVU.MOREX.r3.5HG0521030* | *HvbHLH117* |
| *HORVU.MOREX.r3.2HG0129450* | *HvbHLH37* | *HORVU.MOREX.r3.2HG0204990* | *HvbHLH118* |
| *HORVU.MOREX.r3.3HG0219210* | *HvbHLH38* | *HORVU.MOREX.r3.5HG0512740* | *HvbHLH119* |
| *HORVU.MOREX.r3.4HG0333850* | *HvbHLH39* | *HORVU.MOREX.r3.1HG0072670* | *HvbHLH120* |
| *HORVU.MOREX.r3.1HG0018510* | *HvbHLH40* | *HORVU.MOREX.r3.3HG0305340* | *HvbHLH121* |
| *HORVU.MOREX.r3.7HG0675770* | *HvbHLH41* | *HORVU.MOREX.r3.1HG0021280* | *HvbHLH122* |
| *HORVU.MOREX.r3.4HG0407180* | *HvbHLH42* | *HORVU.MOREX.r3.4HG0392650* | *HvbHLH123* |
| *HORVU.MOREX.r3.4HG0411230* | *HvbHLH43* | *HORVU.MOREX.r3.7HG0666110* | *HvbHLH124* |
| *HORVU.MOREX.r3.5HG0421420* | *HvbHLH44* | *HORVU.MOREX.r3.7HG0671700* | *HvbHLH125* |
| *HORVU.MOREX.r3.7HG0655660* | *HvbHLH45* | *HORVU.MOREX.r3.1HG0080250* | *HvbHLH126* |
| *HORVU.MOREX.r3.3HG0251570* | *HvbHLH46* | *HORVU.MOREX.r3.4HG0403770* | *HvbHLH127* |
| *HORVU.MOREX.r3.2HG0136970* | *HvbHLH47* | *HORVU.MOREX.r3.6HG0603290* | *HvbHLH128* |
| *HORVU.MOREX.r3.2HG0158070* | *HvbHLH48* | *HORVU.MOREX.r3.2HG0208340* | *HvbHLH129* |
| *HORVU.MOREX.r3.2HG0205970* | *HvbHLH49* | *HORVU.MOREX.r3.3HG0321630* | *HvbHLH130* |
| *HORVU.MOREX.r3.2HG0115210* | *HvbHLH50* | *HORVU.MOREX.r3.6HG0558110* | *HvbHLH131* |
| *HORVU.MOREX.r3.2HG0187860* | *HvbHLH51* | *HORVU.MOREX.r3.2HG0177600* | *HvbHLH132* |
| *HORVU.MOREX.r3.5HG0488000* | *HvbHLH52* | *HORVU.MOREX.r3.1HG0073880* | *HvbHLH133* |
| *HORVU.MOREX.r3.6HG0605430* | *HvbHLH53* | *HORVU.MOREX.r3.5HG0495090* | *HvbHLH134* |
| *HORVU.MOREX.r3.4HG0362890* | *HvbHLH54* | *HORVU.MOREX.r3.5HG0433720* | *HvbHLH135* |
| *HORVU.MOREX.r3.3HG0283820* | *HvbHLH55* | *HORVU.MOREX.r3.7HG0658960* | *HvbHLH136* |
| *HORVU.MOREX.r3.7HG0684990* | *HvbHLH56* | *HORVU.MOREX.r3.3HG0312340* | *HvbHLH137* |
| *HORVU.MOREX.r3.5HG0488610* | *HvbHLH57* | *HORVU.MOREX.r3.6HG0611310* | *HvbHLH138* |
| *HORVU.MOREX.r3.1HG0067720* | *HvbHLH58* | *HORVU.MOREX.r3.6HG0545980* | *HvbHLH139* |
| *HORVU.MOREX.r3.3HG0235850* | *HvbHLH59* | *HORVU.MOREX.r3.5HG0509430* | *HvbHLH140* |
| *HORVU.MOREX.r3.6HG0619980* | *HvbHLH60* | *HORVU.MOREX.r3.4HG0363820* | *HvbHLH141* |
| *HORVU.MOREX.r3.3HG0231320* | *HvbHLH61* | *HORVU.MOREX.r3.4HG0339300* | *HvbHLH142* |
| *HORVU.MOREX.r3.4HG0412560* | *HvbHLH62* | *HORVU.MOREX.r3.4HG0343040* | *HvbHLH143* |
| *HORVU.MOREX.r3.7HG0652440* | *HvbHLH63* | *HORVU.MOREX.r3.3HG0321600* | *HvbHLH144* |
| *HORVU.MOREX.r3.6HG0549910* | *HvbHLH64* | *HORVU.MOREX.r3.1HG0025660* | *HvbHLH145* |
| *HORVU.MOREX.r3.2HG0194180* | *HvbHLH65* | *HORVU.MOREX.r3.7HG0663610* | *HvbHLH146* |
| *HORVU.MOREX.r3.6HG0608460* | *HvbHLH66* | *HORVU.MOREX.r3.7HG0703220* | *HvbHLH147* |
| *HORVU.MOREX.r3.6HG0627040* | *HvbHLH67* | *HORVU.MOREX.r3.2HG0197140* | *HvbHLH148* |
| *HORVU.MOREX.r3.3HG0249810* | *HvbHLH68* | *HORVU.MOREX.r3.4HG0403680* | *HvbHLH149* |
| *HORVU.MOREX.r3.2HG0159040* | *HvbHLH69* | *HORVU.MOREX.r3.7HG0713070* | *HvbHLH150* |
| *HORVU.MOREX.r3.1HG0079000* | *HvbHLH70* | *HORVU.MOREX.r3.2HG0160130* | *HvbHLH151* |
| *HORVU.MOREX.r3.2HG0197910* | *HvbHLH71* | *HORVU.MOREX.r3.2HG0107680* | *HvbHLH152* |
| *HORVU.MOREX.r3.3HG0249020* | *HvbHLH72* | *HORVU.MOREX.r3.4HG0407360* | *HvbHLH153* |
| *HORVU.MOREX.r3.4HG0333870* | *HvbHLH73* | *HORVU.MOREX.r3.4HG0394680* | *HvbHLH154* |
| *HORVU.MOREX.r3.3HG0236050* | *HvbHLH74* | *HORVU.MOREX.r3.5HG0487550* | *HvbHLH155* |
| *HORVU.MOREX.r3.5HG0484930* | *HvbHLH75* | *HORVU.MOREX.r3.3HG0312370* | *HvbHLH156* |
| *HORVU.MOREX.r3.6HG0606510* | *HvbHLH76* | *HORVU.MOREX.r3.7HG0642120* | *HvbHLH157* |
| *HORVU.MOREX.r3.4HG0402030* | *HvbHLH77* | *HORVU.MOREX.r3.4HG0406540* | *HvbHLH158* |
| *HORVU.MOREX.r3.2HG0188710* | *HvbHLH78* | *HORVU.MOREX.r3.3HG0266110* | *HvbHLH159* |
| *HORVU.MOREX.r3.5HG0477680* | *HvbHLH79* | *HORVU.MOREX.r3.4HG0415290* | *HvbHLH160* |
| *HORVU.MOREX.r3.5HG0502370* | *HvbHLH80* | *HORVU.MOREX.r3.5HG0430270* | *HvbHLH161* |
| *HORVU.MOREX.r3.5HG0530630* | *HvbHLH81* |  |  |
