# Supplementary material for: Investigating the regulatory role of HvANT2 in anthocyanin biosynthesis through protein–motif interaction in Qingke
Source: PeerJ. 2024 Jul 10;12:e17736. doi: 10.7717/peerj.17736 (PMC11246018; doi:10.7717/peerj.17736)
Supplement: Supplemental Information 4 [file peerj-12-17736-s004.docx]

| **Chromosome** | **Gene ID** | **Chromosome** | **Gene ID** |
| --- | --- | --- | --- |
| Hv--1H | Hv--transcript:HORVU.MOREX.r3.1HG0018510.1 | AT--2 | AT--transcript:AT2G46970.1 |
| Hv--1H | Hv--transcript:HORVU.MOREX.r3.1HG0018510.1 | AT--3 | AT--transcript:AT3G62090.2 |
| Hv--1H | Hv--transcript:HORVU.MOREX.r3.1HG0053170.1 | AT--4 | AT--transcript:AT4G17880.1 |
| Hv--1H | Hv--transcript:HORVU.MOREX.r3.1HG0053170.1 | AT--5 | AT--transcript:AT5G46760.1 |
| Hv--2H | Hv--transcript:HORVU.MOREX.r3.2HG0208340.1 | AT--1 | AT--transcript:AT1G61660.1 |
| Hv--2H | Hv--transcript:HORVU.MOREX.r3.2HG0110130.1 | AT--1 | AT--transcript:AT1G09250.1 |
| Hv--3H | Hv--transcript:HORVU.MOREX.r3.3HG0294110.1 | AT--1 | AT--transcript:AT1G61660.1 |
| Hv--3H | Hv--transcript:HORVU.MOREX.r3.3HG0294110.1 | AT--4 | AT--transcript:AT4G21340.1 |
| Hv--4H | Hv--transcript:HORVU.MOREX.r3.4HG0394410.1 | AT--4 | AT--transcript:AT4G37850.2 |
| Hv--4H | Hv--transcript:HORVU.MOREX.r3.4HG0398470.1 | AT--5 | AT--transcript:AT5G43175.1 |
| Hv--4H | Hv--transcript:HORVU.MOREX.r3.4HG0338770.1 | AT--5 | AT--transcript:AT5G65640.1 |
| Hv--5H | Hv--transcript:HORVU.MOREX.r3.5HG0490730.1 | AT--2 | AT--transcript:AT2G42280.3 |
| Hv--6H | Hv--transcript:HORVU.MOREX.r3.6HG0615060.1 | AT--1 | AT--transcript:AT1G74500.1 |
| Hv--6H | Hv--transcript:HORVU.MOREX.r3.6HG0607570.1 | AT--2 | AT--transcript:AT2G18300.3 |
| Hv--6H | Hv--transcript:HORVU.MOREX.r3.6HG0607570.1 | AT--4 | AT--transcript:AT4G36540.1 |
| Hv--6H | Hv--transcript:HORVU.MOREX.r3.6HG0605430.1 | AT--5 | AT--transcript:AT5G46690.1 |
| Hv--7H | Hv--transcript:HORVU.MOREX.r3.7HG0684990.1 | AT--5 | AT--transcript:AT5G50915.2 |
| Hv--7H | Hv--transcript:HORVU.MOREX.r3.7HG0658960.1 | AT--5 | AT--transcript:AT5G58010.1 |
| Hv--1H | Hv--transcript:HORVU.MOREX.r3.1HG0080250.1 | Os--1 | Os--transcript:Os01t0707500-01 |
| Hv--1H | Hv--transcript:HORVU.MOREX.r3.1HG0025660.1 | Os--1 | Os--transcript:Os01t0159800-02 |
| Hv--1H | Hv--transcript:HORVU.MOREX.r3.1HG0018510.1 | Os--1 | Os--transcript:Os01t0286100-01 |
| Hv--1H | Hv--transcript:HORVU.MOREX.r3.1HG0056640.1 | Os--10 | Os--transcript:Os10t0556200-01 |
| Hv--1H | Hv--transcript:HORVU.MOREX.r3.1HG0053170.1 | Os--10 | Os--transcript:Os10t0575000-01 |
| Hv--1H | Hv--transcript:HORVU.MOREX.r3.1HG0018510.1 | Os--5 | Os--transcript:Os05t0139100-01 |
| Hv--1H | Hv--transcript:HORVU.MOREX.r3.1HG0025660.1 | Os--5 | Os--transcript:Os05t0163900-01 |
| Hv--1H | Hv--transcript:HORVU.MOREX.r3.1HG0032650.1 | Os--5 | Os--transcript:Os05t0228400-01 |
| Hv--1H | Hv--transcript:HORVU.MOREX.r3.1HG0073880.1 | Os--5 | Os--transcript:Os05t0455400-01 |
| Hv--1H | Hv--transcript:HORVU.MOREX.r3.1HG0080250.1 | Os--5 | Os--transcript:Os05t0541400-01 |
| Hv--2H | Hv--transcript:HORVU.MOREX.r3.2HG0197140.1 | Os--2 | Os--transcript:Os02t0691500-01 |
| Hv--2H | Hv--transcript:HORVU.MOREX.r3.2HG0187860.1 | Os--2 | Os--transcript:Os02t0671300-01 |
| Hv--2H | Hv--transcript:HORVU.MOREX.r3.2HG0110130.1 | Os--3 | Os--transcript:Os03t0311600-01 |
| Hv--2H | Hv--transcript:HORVU.MOREX.r3.2HG0122430.1 | Os--3 | Os--transcript:Os03t0379300-01 |
| Hv--2H | Hv--transcript:HORVU.MOREX.r3.2HG0153790.1 | Os--3 | Os--transcript:Os03t0782500-01 |
| Hv--2H | Hv--transcript:HORVU.MOREX.r3.2HG0161630.1 | Os--4 | Os--transcript:Os04t0381700-00 |
| Hv--2H | Hv--transcript:HORVU.MOREX.r3.2HG0197140.1 | Os--4 | Os--transcript:Os04t0590800-01 |
| Hv--2H | Hv--transcript:HORVU.MOREX.r3.2HG0158950.1 | Os--4 | Os--transcript:Os04t0300600-00 |
| Hv--2H | Hv--transcript:HORVU.MOREX.r3.2HG0205970.1 | Os--4 | Os--transcript:Os04t0641700-00 |
| Hv--2H | Hv--transcript:HORVU.MOREX.r3.2HG0208340.1 | Os--4 | Os--transcript:Os04t0631600-01 |
| Hv--2H | Hv--transcript:HORVU.MOREX.r3.2HG0187860.1 | Os--4 | Os--transcript:Os04t0565900-01 |
| Hv--2H | Hv--transcript:HORVU.MOREX.r3.2HG0188710.1 | Os--4 | Os--transcript:Os04t0557200-01 |
| Hv--2H | Hv--transcript:HORVU.MOREX.r3.2HG0194180.1 | Os--4 | Os--transcript:Os04t0599300-01 |
| Hv--2H | Hv--transcript:HORVU.MOREX.r3.2HG0177600.1 | Os--4 | Os--transcript:Os04t0493100-01 |
| Hv--2H | Hv--transcript:HORVU.MOREX.r3.2HG0122430.1 | Os--7 | Os--transcript:Os07t0628500-02 |
| Hv--2H | Hv--transcript:HORVU.MOREX.r3.2HG0136970.1 | Os--7 | Os--transcript:Os07t0549600-01 |
| Hv--2H | Hv--transcript:HORVU.MOREX.r3.2HG0138530.1 | Os--7 | Os--transcript:Os07t0543000-01 |
| Hv--2H | Hv--transcript:HORVU.MOREX.r3.2HG0153790.1 | Os--7 | Os--transcript:Os07t0143200-01 |
| Hv--2H | Hv--transcript:HORVU.MOREX.r3.2HG0158070.1 | Os--7 | Os--transcript:Os07t0101300-01 |
| Hv--2H | Hv--transcript:HORVU.MOREX.r3.2HG0208340.1 | Os--8 | Os--transcript:Os08t0179400-00 |
| Hv--3H | Hv--transcript:HORVU.MOREX.r3.3HG0266110.1 | Os--1 | Os--transcript:Os01t0566800-00 |
| Hv--3H | Hv--transcript:HORVU.MOREX.r3.3HG0267290.1 | Os--1 | Os--transcript:Os01t0575200-01 |
| Hv--3H | Hv--transcript:HORVU.MOREX.r3.3HG0283700.1 | Os--1 | Os--transcript:Os01t0705700-01 |
| Hv--3H | Hv--transcript:HORVU.MOREX.r3.3HG0283820.1 | Os--1 | Os--transcript:Os01t0707500-01 |
| Hv--3H | Hv--transcript:HORVU.MOREX.r3.3HG0294110.1 | Os--1 | Os--transcript:Os01t0784900-00 |
| Hv--3H | Hv--transcript:HORVU.MOREX.r3.3HG0249020.1 | Os--1 | Os--transcript:Os01t0230200-01 |
| Hv--3H | Hv--transcript:HORVU.MOREX.r3.3HG0249810.1 | Os--1 | Os--transcript:Os01t0235700-02 |
| Hv--3H | Hv--transcript:HORVU.MOREX.r3.3HG0251570.1 | Os--1 | Os--transcript:Os01t0243400-01 |
| Hv--3H | Hv--transcript:HORVU.MOREX.r3.3HG0309530.1 | Os--1 | Os--transcript:Os01t0915600-01 |
| Hv--3H | Hv--transcript:HORVU.MOREX.r3.3HG0312340.1 | Os--1 | Os--transcript:Os01t0928000-01 |
| Hv--3H | Hv--transcript:HORVU.MOREX.r3.3HG0219210.1 | Os--1 | Os--transcript:Os01t0111500-01 |
| Hv--3H | Hv--transcript:HORVU.MOREX.r3.3HG0236050.1 | Os--1 | Os--transcript:Os01t0195801-00 |
| Hv--3H | Hv--transcript:HORVU.MOREX.r3.3HG0305340.1 | Os--1 | Os--transcript:Os01t0900800-01 |
| Hv--3H | Hv--transcript:HORVU.MOREX.r3.3HG0235850.1 | Os--1 | Os--transcript:Os01t0159800-02 |
| Hv--3H | Hv--transcript:HORVU.MOREX.r3.3HG0244970.1 | Os--1 | Os--transcript:Os01t0218100-01 |
| Hv--3H | Hv--transcript:HORVU.MOREX.r3.3HG0231320.1 | Os--1 | Os--transcript:Os01t0105700-01 |
| Hv--3H | Hv--transcript:HORVU.MOREX.r3.3HG0249020.1 | Os--5 | Os--transcript:Os05t0228400-01 |
| Hv--3H | Hv--transcript:HORVU.MOREX.r3.3HG0283820.1 | Os--5 | Os--transcript:Os05t0541400-01 |
| Hv--3H | Hv--transcript:HORVU.MOREX.r3.3HG0236050.1 | Os--5 | Os--transcript:Os05t0199800-01 |
| Hv--4H | Hv--transcript:HORVU.MOREX.r3.4HG0403770.1 | Os--10 | Os--transcript:Os10t0403800-00 |
| Hv--4H | Hv--transcript:HORVU.MOREX.r3.4HG0394410.1 | Os--10 | Os--transcript:Os10t0104300-00 |
| Hv--4H | Hv--transcript:HORVU.MOREX.r3.4HG0350240.1 | Os--11 | Os--transcript:Os11t0158500-01 |
| Hv--4H | Hv--transcript:HORVU.MOREX.r3.4HG0333850.1 | Os--12 | Os--transcript:Os12t0632600-00 |
| Hv--4H | Hv--transcript:HORVU.MOREX.r3.4HG0343040.1 | Os--12 | Os--transcript:Os12t0589000-00 |
| Hv--4H | Hv--transcript:HORVU.MOREX.r3.4HG0350240.1 | Os--12 | Os--transcript:Os12t0160400-01 |
| Hv--4H | Hv--transcript:HORVU.MOREX.r3.4HG0333870.1 | Os--3 | Os--transcript:Os03t0671000-00 |
| Hv--4H | Hv--transcript:HORVU.MOREX.r3.4HG0339300.1 | Os--3 | Os--transcript:Os03t0728900-00 |
| Hv--4H | Hv--transcript:HORVU.MOREX.r3.4HG0403680.1 | Os--3 | Os--transcript:Os03t0171300-01 |
| Hv--4H | Hv--transcript:HORVU.MOREX.r3.4HG0406540.1 | Os--3 | Os--transcript:Os03t0188400-01 |
| Hv--4H | Hv--transcript:HORVU.MOREX.r3.4HG0394410.1 | Os--3 | Os--transcript:Os03t0229100-00 |
| Hv--4H | Hv--transcript:HORVU.MOREX.r3.4HG0394680.1 | Os--3 | Os--transcript:Os03t0231950-01 |
| Hv--4H | Hv--transcript:HORVU.MOREX.r3.4HG0394410.1 | Os--3 | Os--transcript:Os03t0725800-01 |
| Hv--4H | Hv--transcript:HORVU.MOREX.r3.4HG0394680.1 | Os--3 | Os--transcript:Os03t0728900-00 |
| Hv--4H | Hv--transcript:HORVU.MOREX.r3.4HG0384570.1 | Os--3 | Os--transcript:Os03t0293400-01 |
| Hv--4H | Hv--transcript:HORVU.MOREX.r3.4HG0387050.1 | Os--3 | Os--transcript:Os03t0279500-00 |
| Hv--4H | Hv--transcript:HORVU.MOREX.r3.4HG0390530.1 | Os--3 | Os--transcript:Os03t0260600-01 |
| Hv--4H | Hv--transcript:HORVU.MOREX.r3.4HG0398470.1 | Os--3 | Os--transcript:Os03t0205300-00 |
| Hv--4H | Hv--transcript:HORVU.MOREX.r3.4HG0409010.1 | Os--3 | Os--transcript:Os03t0135700-01 |
| Hv--4H | Hv--transcript:HORVU.MOREX.r3.4HG0412560.1 | Os--3 | Os--transcript:Os03t0122100-00 |
| Hv--4H | Hv--transcript:HORVU.MOREX.r3.4HG0406540.1 | Os--9 | Os--transcript:Os09t0468700-00 |
| Hv--5H | Hv--transcript:HORVU.MOREX.r3.5HG0458030.1 | Os--11 | Os--transcript:Os11t0158500-01 |
| Hv--5H | Hv--transcript:HORVU.MOREX.r3.5HG0438460.1 | Os--12 | Os--transcript:Os12t0589000-00 |
| Hv--5H | Hv--transcript:HORVU.MOREX.r3.5HG0430270.1 | Os--12 | Os--transcript:Os12t0610200-01 |
| Hv--5H | Hv--transcript:HORVU.MOREX.r3.5HG0458030.1 | Os--12 | Os--transcript:Os12t0160400-01 |
| Hv--5H | Hv--transcript:HORVU.MOREX.r3.5HG0488610.1 | Os--2 | Os--transcript:Os02t0726700-01 |
| Hv--5H | Hv--transcript:HORVU.MOREX.r3.5HG0509430.1 | Os--3 | Os--transcript:Os03t0741100-01 |
| Hv--5H | Hv--transcript:HORVU.MOREX.r3.5HG0512260.1 | Os--3 | Os--transcript:Os03t0759700-01 |
| Hv--5H | Hv--transcript:HORVU.MOREX.r3.5HG0515900.1 | Os--3 | Os--transcript:Os03t0782500-01 |
| Hv--5H | Hv--transcript:HORVU.MOREX.r3.5HG0519660.1 | Os--3 | Os--transcript:Os03t0797600-01 |
| Hv--5H | Hv--transcript:HORVU.MOREX.r3.5HG0521030.1 | Os--3 | Os--transcript:Os03t0802900-01 |
| Hv--5H | Hv--transcript:HORVU.MOREX.r3.5HG0524270.1 | Os--3 | Os--transcript:Os03t0811400-00 |
| Hv--5H | Hv--transcript:HORVU.MOREX.r3.5HG0488000.1 | Os--3 | Os--transcript:Os03t0188400-01 |
| Hv--5H | Hv--transcript:HORVU.MOREX.r3.5HG0519660.1 | Os--7 | Os--transcript:Os07t0182200-01 |
| Hv--5H | Hv--transcript:HORVU.MOREX.r3.5HG0521030.1 | Os--7 | Os--transcript:Os07t0193800-01 |
| Hv--5H | Hv--transcript:HORVU.MOREX.r3.5HG0515900.1 | Os--7 | Os--transcript:Os07t0143200-01 |
| Hv--5H | Hv--transcript:HORVU.MOREX.r3.5HG0487550.1 | Os--8 | Os--transcript:Os08t0477900-00 |
| Hv--5H | Hv--transcript:HORVU.MOREX.r3.5HG0488610.1 | Os--8 | Os--transcript:Os08t0490000-01 |
| Hv--5H | Hv--transcript:HORVU.MOREX.r3.5HG0490730.1 | Os--8 | Os--transcript:Os08t0506700-01 |
| Hv--5H | Hv--transcript:HORVU.MOREX.r3.5HG0493040.1 | Os--8 | Os--transcript:Os08t0524800-01 |
| Hv--5H | Hv--transcript:HORVU.MOREX.r3.5HG0495090.1 | Os--8 | Os--transcript:Os08t0536800-01 |
| Hv--5H | Hv--transcript:HORVU.MOREX.r3.5HG0484930.1 | Os--8 | Os--transcript:Os08t0432800-01 |
| Hv--5H | Hv--transcript:HORVU.MOREX.r3.5HG0486320.1 | Os--9 | Os--transcript:Os09t0455300-01 |
| Hv--5H | Hv--transcript:HORVU.MOREX.r3.5HG0487550.1 | Os--9 | Os--transcript:Os09t0463900-00 |
| Hv--5H | Hv--transcript:HORVU.MOREX.r3.5HG0488000.1 | Os--9 | Os--transcript:Os09t0468700-00 |
| Hv--5H | Hv--transcript:HORVU.MOREX.r3.5HG0488610.1 | Os--9 | Os--transcript:Os09t0475400-01 |
| Hv--5H | Hv--transcript:HORVU.MOREX.r3.5HG0490730.1 | Os--9 | Os--transcript:Os09t0487900-01 |
| Hv--5H | Hv--transcript:HORVU.MOREX.r3.5HG0493040.1 | Os--9 | Os--transcript:Os09t0501600-01 |
| Hv--5H | Hv--transcript:HORVU.MOREX.r3.5HG0495090.1 | Os--9 | Os--transcript:Os09t0510500-01 |
| Hv--5H | Hv--transcript:HORVU.MOREX.r3.5HG0484090.1 | Os--9 | Os--transcript:Os09t0417400-00 |
| Hv--5H | Hv--transcript:HORVU.MOREX.r3.5HG0484930.1 | Os--9 | Os--transcript:Os09t0410700-00 |
| Hv--6H | Hv--transcript:HORVU.MOREX.r3.6HG0592420.1 | Os--2 | Os--transcript:Os02t0603600-01 |
| Hv--6H | Hv--transcript:HORVU.MOREX.r3.6HG0603090.1 | Os--2 | Os--transcript:Os02t0671300-01 |
| Hv--6H | Hv--transcript:HORVU.MOREX.r3.6HG0605430.1 | Os--2 | Os--transcript:Os02t0691500-01 |
| Hv--6H | Hv--transcript:HORVU.MOREX.r3.6HG0607570.1 | Os--2 | Os--transcript:Os02t0705500-02 |
| Hv--6H | Hv--transcript:HORVU.MOREX.r3.6HG0608460.1 | Os--2 | Os--transcript:Os02t0710300-01 |
| Hv--6H | Hv--transcript:HORVU.MOREX.r3.6HG0611310.1 | Os--2 | Os--transcript:Os02t0726700-01 |
| Hv--6H | Hv--transcript:HORVU.MOREX.r3.6HG0624240.1 | Os--2 | Os--transcript:Os02t0795800-00 |
| Hv--6H | Hv--transcript:HORVU.MOREX.r3.6HG0627040.1 | Os--2 | Os--transcript:Os02t0805250-01 |
| Hv--6H | Hv--transcript:HORVU.MOREX.r3.6HG0580040.1 | Os--2 | Os--transcript:Os02t0221100-00 |
| Hv--6H | Hv--transcript:HORVU.MOREX.r3.6HG0545980.1 | Os--2 | Os--transcript:Os02t0116600-01 |
| Hv--6H | Hv--transcript:HORVU.MOREX.r3.6HG0615060.1 | Os--2 | Os--transcript:Os02t0747900-01 |
| Hv--6H | Hv--transcript:HORVU.MOREX.r3.6HG0549910.1 | Os--2 | Os--transcript:Os02t0120500-02 |
| Hv--6H | Hv--transcript:HORVU.MOREX.r3.6HG0592420.1 | Os--4 | Os--transcript:Os04t0489600-02 |
| Hv--6H | Hv--transcript:HORVU.MOREX.r3.6HG0603090.1 | Os--4 | Os--transcript:Os04t0565900-01 |
| Hv--6H | Hv--transcript:HORVU.MOREX.r3.6HG0605430.1 | Os--4 | Os--transcript:Os04t0590800-01 |
| Hv--6H | Hv--transcript:HORVU.MOREX.r3.6HG0606510.1 | Os--4 | Os--transcript:Os04t0599300-01 |
| Hv--6H | Hv--transcript:HORVU.MOREX.r3.6HG0615060.1 | Os--6 | Os--transcript:Os06t0226500-01 |
| Hv--6H | Hv--transcript:HORVU.MOREX.r3.6HG0580040.1 | Os--6 | Os--transcript:Os06t0570900-00 |
| Hv--6H | Hv--transcript:HORVU.MOREX.r3.6HG0624240.1 | Os--6 | Os--transcript:Os06t0184000-00 |
| Hv--6H | Hv--transcript:HORVU.MOREX.r3.6HG0627040.1 | Os--6 | Os--transcript:Os06t0164400-01 |
| Hv--6H | Hv--transcript:HORVU.MOREX.r3.6HG0611310.1 | Os--9 | Os--transcript:Os09t0475400-01 |
| Hv--7H | Hv--transcript:HORVU.MOREX.r3.7HG0682660.1 | Os--1 | Os--transcript:Os01t0915600-01 |
| Hv--7H | Hv--transcript:HORVU.MOREX.r3.7HG0716830.1 | Os--2 | Os--transcript:Os02t0221100-00 |
| Hv--7H | Hv--transcript:HORVU.MOREX.r3.7HG0675770.1 | Os--2 | Os--transcript:Os02t0710300-01 |
| Hv--7H | Hv--transcript:HORVU.MOREX.r3.7HG0666110.1 | Os--2 | Os--transcript:Os02t0747900-01 |
| Hv--7H | Hv--transcript:HORVU.MOREX.r3.7HG0708530.1 | Os--2 | Os--transcript:Os02t0257500-00 |
| Hv--7H | Hv--transcript:HORVU.MOREX.r3.7HG0658960.1 | Os--2 | Os--transcript:Os02t0795800-00 |
| Hv--7H | Hv--transcript:HORVU.MOREX.r3.7HG0655660.1 | Os--6 | Os--transcript:Os06t0164400-01 |
| Hv--7H | Hv--transcript:HORVU.MOREX.r3.7HG0658960.1 | Os--6 | Os--transcript:Os06t0184000-00 |
| Hv--7H | Hv--transcript:HORVU.MOREX.r3.7HG0660920.1 | Os--6 | Os--transcript:Os06t0193400-01 |
| Hv--7H | Hv--transcript:HORVU.MOREX.r3.7HG0666110.1 | Os--6 | Os--transcript:Os06t0226500-01 |
| Hv--7H | Hv--transcript:HORVU.MOREX.r3.7HG0671700.1 | Os--6 | Os--transcript:Os06t0275600-01 |
| Hv--7H | Hv--transcript:HORVU.MOREX.r3.7HG0708530.1 | Os--6 | Os--transcript:Os06t0526100-01 |
| Hv--7H | Hv--transcript:HORVU.MOREX.r3.7HG0675770.1 | Os--6 | Os--transcript:Os06t0496400-01 |
| Hv--7H | Hv--transcript:HORVU.MOREX.r3.7HG0716830.1 | Os--6 | Os--transcript:Os06t0570900-00 |
| Hv--7H | Hv--transcript:HORVU.MOREX.r3.7HG0724770.1 | Os--6 | Os--transcript:Os06t0613500-01 |
| Hv--7H | Hv--transcript:HORVU.MOREX.r3.7HG0682660.1 | Os--8 | Os--transcript:Os08t0524800-01 |
| Hv--7H | Hv--transcript:HORVU.MOREX.r3.7HG0684990.1 | Os--8 | Os--transcript:Os08t0536800-01 |
| Hv--7H | Hv--transcript:HORVU.MOREX.r3.7HG0702010.1 | Os--8 | Os--transcript:Os08t0138500-01 |
| Hv--7H | Hv--transcript:HORVU.MOREX.r3.7HG0706460.1 | Os--8 | Os--transcript:Os08t0108500-01 |
| Hv--7H | Hv--transcript:HORVU.MOREX.r3.7HG0679760.1 | Os--9 | Os--transcript:Os09t0487900-01 |
| Hv--7H | Hv--transcript:HORVU.MOREX.r3.7HG0682660.1 | Os--9 | Os--transcript:Os09t0501600-01 |
| Hv--7H | Hv--transcript:HORVU.MOREX.r3.7HG0684990.1 | Os--9 | Os--transcript:Os09t0510500-01 |
| Hv--7H | Hv--transcript:HORVU.MOREX.r3.7HG0671700.1 | Os--9 | Os--transcript:Os09t0474100-01 |
| Hv--7H | Hv--transcript:HORVU.MOREX.r3.7HG0663610.1 | Os--9 | Os--transcript:Os09t0455300-01 |
| Hv--1H | Hv--transcript:HORVU.MOREX.r3.1HG0056640.1 | Zm--1 | Zm--transcript:Zm00001eb023350_T002 |
| Hv--1H | Hv--transcript:HORVU.MOREX.r3.1HG0053170.1 | Zm--1 | Zm--transcript:Zm00001eb024330_T001 |
| Hv--1H | Hv--transcript:HORVU.MOREX.r3.1HG0018510.1 | Zm--10 | Zm--transcript:Zm00001eb417610_T002 |
| Hv--1H | Hv--transcript:HORVU.MOREX.r3.1HG0067720.1 | Zm--3 | Zm--transcript:Zm00001eb151270_T003 |
| Hv--1H | Hv--transcript:HORVU.MOREX.r3.1HG0025660.1 | Zm--3 | Zm--transcript:Zm00001eb123570_T003 |
| Hv--1H | Hv--transcript:HORVU.MOREX.r3.1HG0080250.1 | Zm--3 | Zm--transcript:Zm00001eb155300_T001 |
| Hv--1H | Hv--transcript:HORVU.MOREX.r3.1HG0018510.1 | Zm--3 | Zm--transcript:Zm00001eb129520_T001 |
| Hv--1H | Hv--transcript:HORVU.MOREX.r3.1HG0067720.1 | Zm--6 | Zm--transcript:Zm00001eb291960_T001 |
| Hv--1H | Hv--transcript:HORVU.MOREX.r3.1HG0073880.1 | Zm--6 | Zm--transcript:Zm00001eb289490_T001 |
| Hv--1H | Hv--transcript:HORVU.MOREX.r3.1HG0080250.1 | Zm--6 | Zm--transcript:Zm00001eb294390_T001 |
| Hv--1H | Hv--transcript:HORVU.MOREX.r3.1HG0025660.1 | Zm--6 | Zm--transcript:Zm00001eb282210_T002 |
| Hv--1H | Hv--transcript:HORVU.MOREX.r3.1HG0018510.1 | Zm--8 | Zm--transcript:Zm00001eb332400_T001 |
| Hv--1H | Hv--transcript:HORVU.MOREX.r3.1HG0032650.1 | Zm--8 | Zm--transcript:Zm00001eb350430_T002 |
| Hv--1H | Hv--transcript:HORVU.MOREX.r3.1HG0053170.1 | Zm--9 | Zm--transcript:Zm00001eb390330_T002 |
| Hv--2H | Hv--transcript:HORVU.MOREX.r3.2HG0110130.1 | Zm--1 | Zm--transcript:Zm00001eb014980_T001 |
| Hv--2H | Hv--transcript:HORVU.MOREX.r3.2HG0122430.1 | Zm--1 | Zm--transcript:Zm00001eb018460_T001 |
| Hv--2H | Hv--transcript:HORVU.MOREX.r3.2HG0161630.1 | Zm--10 | Zm--transcript:Zm00001eb420910_T001 |
| Hv--2H | Hv--transcript:HORVU.MOREX.r3.2HG0187860.1 | Zm--10 | Zm--transcript:Zm00001eb429090_T001 |
| Hv--2H | Hv--transcript:HORVU.MOREX.r3.2HG0188710.1 | Zm--10 | Zm--transcript:Zm00001eb429330_T001 |
| Hv--2H | Hv--transcript:HORVU.MOREX.r3.2HG0197140.1 | Zm--10 | Zm--transcript:Zm00001eb428400_T001 |
| Hv--2H | Hv--transcript:HORVU.MOREX.r3.2HG0205970.1 | Zm--10 | Zm--transcript:Zm00001eb431920_T001 |
| Hv--2H | Hv--transcript:HORVU.MOREX.r3.2HG0205970.1 | Zm--2 | Zm--transcript:Zm00001eb068900_T001 |
| Hv--2H | Hv--transcript:HORVU.MOREX.r3.2HG0208340.1 | Zm--2 | Zm--transcript:Zm00001eb069700_T002 |
| Hv--2H | Hv--transcript:HORVU.MOREX.r3.2HG0188710.1 | Zm--2 | Zm--transcript:Zm00001eb074320_T001 |
| Hv--2H | Hv--transcript:HORVU.MOREX.r3.2HG0177600.1 | Zm--2 | Zm--transcript:Zm00001eb078760_T001 |
| Hv--2H | Hv--transcript:HORVU.MOREX.r3.2HG0161630.1 | Zm--2 | Zm--transcript:Zm00001eb085690_T001 |
| Hv--2H | Hv--transcript:HORVU.MOREX.r3.2HG0197140.1 | Zm--2 | Zm--transcript:Zm00001eb072400_T001 |
| Hv--2H | Hv--transcript:HORVU.MOREX.r3.2HG0136970.1 | Zm--2 | Zm--transcript:Zm00001eb106620_T001 |
| Hv--2H | Hv--transcript:HORVU.MOREX.r3.2HG0197140.1 | Zm--5 | Zm--transcript:Zm00001eb251200_T003 |
| Hv--2H | Hv--transcript:HORVU.MOREX.r3.2HG0187860.1 | Zm--5 | Zm--transcript:Zm00001eb250230_T002 |
| Hv--2H | Hv--transcript:HORVU.MOREX.r3.2HG0194180.1 | Zm--5 | Zm--transcript:Zm00001eb251650_T003 |
| Hv--2H | Hv--transcript:HORVU.MOREX.r3.2HG0122430.1 | Zm--7 | Zm--transcript:Zm00001eb328250_T003 |
| Hv--2H | Hv--transcript:HORVU.MOREX.r3.2HG0138530.1 | Zm--7 | Zm--transcript:Zm00001eb323440_T004 |
| Hv--2H | Hv--transcript:HORVU.MOREX.r3.2HG0158070.1 | Zm--7 | Zm--transcript:Zm00001eb298040_T002 |
| Hv--2H | Hv--transcript:HORVU.MOREX.r3.2HG0110130.1 | Zm--7 | Zm--transcript:Zm00001eb331270_T001 |
| Hv--3H | Hv--transcript:HORVU.MOREX.r3.3HG0236050.1 | Zm--3 | Zm--transcript:Zm00001eb120960_T001 |
| Hv--3H | Hv--transcript:HORVU.MOREX.r3.3HG0305340.1 | Zm--3 | Zm--transcript:Zm00001eb144450_T002 |
| Hv--3H | Hv--transcript:HORVU.MOREX.r3.3HG0266110.1 | Zm--3 | Zm--transcript:Zm00001eb159960_T005 |
| Hv--3H | Hv--transcript:HORVU.MOREX.r3.3HG0267290.1 | Zm--3 | Zm--transcript:Zm00001eb160210_T001 |
| Hv--3H | Hv--transcript:HORVU.MOREX.r3.3HG0235850.1 | Zm--3 | Zm--transcript:Zm00001eb123570_T003 |
| Hv--3H | Hv--transcript:HORVU.MOREX.r3.3HG0244970.1 | Zm--3 | Zm--transcript:Zm00001eb119900_T001 |
| Hv--3H | Hv--transcript:HORVU.MOREX.r3.3HG0231320.1 | Zm--3 | Zm--transcript:Zm00001eb126840_T001 |
| Hv--3H | Hv--transcript:HORVU.MOREX.r3.3HG0283700.1 | Zm--3 | Zm--transcript:Zm00001eb155410_T001 |
| Hv--3H | Hv--transcript:HORVU.MOREX.r3.3HG0283820.1 | Zm--3 | Zm--transcript:Zm00001eb155300_T001 |
| Hv--3H | Hv--transcript:HORVU.MOREX.r3.3HG0294110.1 | Zm--3 | Zm--transcript:Zm00001eb151270_T003 |
| Hv--3H | Hv--transcript:HORVU.MOREX.r3.3HG0312340.1 | Zm--3 | Zm--transcript:Zm00001eb142500_T001 |
| Hv--3H | Hv--transcript:HORVU.MOREX.r3.3HG0249020.1 | Zm--3 | Zm--transcript:Zm00001eb119270_T003 |
| Hv--3H | Hv--transcript:HORVU.MOREX.r3.3HG0249810.1 | Zm--3 | Zm--transcript:Zm00001eb119000_T002 |
| Hv--3H | Hv--transcript:HORVU.MOREX.r3.3HG0321590.1 | Zm--3 | Zm--transcript:Zm00001eb140680_T001 |
| Hv--3H | Hv--transcript:HORVU.MOREX.r3.3HG0236050.1 | Zm--6 | Zm--transcript:Zm00001eb284130_T001 |
| Hv--3H | Hv--transcript:HORVU.MOREX.r3.3HG0249020.1 | Zm--6 | Zm--transcript:Zm00001eb284290_T001 |
| Hv--3H | Hv--transcript:HORVU.MOREX.r3.3HG0283820.1 | Zm--6 | Zm--transcript:Zm00001eb294390_T001 |
| Hv--3H | Hv--transcript:HORVU.MOREX.r3.3HG0294110.1 | Zm--6 | Zm--transcript:Zm00001eb291960_T001 |
| Hv--3H | Hv--transcript:HORVU.MOREX.r3.3HG0266110.1 | Zm--8 | Zm--transcript:Zm00001eb356930_T002 |
| Hv--3H | Hv--transcript:HORVU.MOREX.r3.3HG0249810.1 | Zm--8 | Zm--transcript:Zm00001eb339360_T001 |
| Hv--3H | Hv--transcript:HORVU.MOREX.r3.3HG0219210.1 | Zm--8 | Zm--transcript:Zm00001eb334500_T001 |
| Hv--3H | Hv--transcript:HORVU.MOREX.r3.3HG0321590.1 | Zm--8 | Zm--transcript:Zm00001eb362800_T001 |
| Hv--3H | Hv--transcript:HORVU.MOREX.r3.3HG0267290.1 | Zm--8 | Zm--transcript:Zm00001eb357120_T002 |
| Hv--3H | Hv--transcript:HORVU.MOREX.r3.3HG0236050.1 | Zm--8 | Zm--transcript:Zm00001eb337860_T002 |
| Hv--3H | Hv--transcript:HORVU.MOREX.r3.3HG0249020.1 | Zm--8 | Zm--transcript:Zm00001eb350430_T002 |
| Hv--4H | Hv--transcript:HORVU.MOREX.r3.4HG0338770.1 | Zm--1 | Zm--transcript:Zm00001eb055830_T002 |
| Hv--4H | Hv--transcript:HORVU.MOREX.r3.4HG0339300.1 | Zm--1 | Zm--transcript:Zm00001eb055990_T001 |
| Hv--4H | Hv--transcript:HORVU.MOREX.r3.4HG0403680.1 | Zm--1 | Zm--transcript:Zm00001eb005500_T001 |
| Hv--4H | Hv--transcript:HORVU.MOREX.r3.4HG0406540.1 | Zm--1 | Zm--transcript:Zm00001eb006680_T002 |
| Hv--4H | Hv--transcript:HORVU.MOREX.r3.4HG0398470.1 | Zm--1 | Zm--transcript:Zm00001eb007580_T001 |
| Hv--4H | Hv--transcript:HORVU.MOREX.r3.4HG0384570.1 | Zm--1 | Zm--transcript:Zm00001eb013510_T002 |
| Hv--4H | Hv--transcript:HORVU.MOREX.r3.4HG0387050.1 | Zm--1 | Zm--transcript:Zm00001eb012680_T002 |
| Hv--4H | Hv--transcript:HORVU.MOREX.r3.4HG0390530.1 | Zm--1 | Zm--transcript:Zm00001eb011370_T001 |
| Hv--4H | Hv--transcript:HORVU.MOREX.r3.4HG0394410.1 | Zm--1 | Zm--transcript:Zm00001eb009610_T001 |
| Hv--4H | Hv--transcript:HORVU.MOREX.r3.4HG0394680.1 | Zm--1 | Zm--transcript:Zm00001eb009490_T010 |
| Hv--4H | Hv--transcript:HORVU.MOREX.r3.4HG0409010.1 | Zm--1 | Zm--transcript:Zm00001eb002830_T001 |
| Hv--4H | Hv--transcript:HORVU.MOREX.r3.4HG0412560.1 | Zm--1 | Zm--transcript:Zm00001eb001630_T002 |
| Hv--4H | Hv--transcript:HORVU.MOREX.r3.4HG0333850.1 | Zm--1 | Zm--transcript:Zm00001eb052150_T001 |
| Hv--4H | Hv--transcript:HORVU.MOREX.r3.4HG0350240.1 | Zm--10 | Zm--transcript:Zm00001eb407240_T001 |
| Hv--4H | Hv--transcript:HORVU.MOREX.r3.4HG0350240.1 | Zm--2 | Zm--transcript:Zm00001eb092180_T001 |
| Hv--4H | Hv--transcript:HORVU.MOREX.r3.4HG0350240.1 | Zm--3 | Zm--transcript:Zm00001eb139520_T002 |
| Hv--4H | Hv--transcript:HORVU.MOREX.r3.4HG0350240.1 | Zm--4 | Zm--transcript:Zm00001eb202130_T002 |
| Hv--4H | Hv--transcript:HORVU.MOREX.r3.4HG0338770.1 | Zm--5 | Zm--transcript:Zm00001eb215740_T001 |
| Hv--4H | Hv--transcript:HORVU.MOREX.r3.4HG0339300.1 | Zm--5 | Zm--transcript:Zm00001eb215610_T004 |
| Hv--4H | Hv--transcript:HORVU.MOREX.r3.4HG0390530.1 | Zm--9 | Zm--transcript:Zm00001eb398190_T001 |
| Hv--4H | Hv--transcript:HORVU.MOREX.r3.4HG0394680.1 | Zm--9 | Zm--transcript:Zm00001eb399390_T002 |
| Hv--4H | Hv--transcript:HORVU.MOREX.r3.4HG0398470.1 | Zm--9 | Zm--transcript:Zm00001eb400660_T002 |
| Hv--4H | Hv--transcript:HORVU.MOREX.r3.4HG0412560.1 | Zm--9 | Zm--transcript:Zm00001eb403770_T001 |
| Hv--4H | Hv--transcript:HORVU.MOREX.r3.4HG0403770.1 | Zm--9 | Zm--transcript:Zm00001eb402170_T001 |
| Hv--4H | Hv--transcript:HORVU.MOREX.r3.4HG0406540.1 | Zm--9 | Zm--transcript:Zm00001eb401480_T001 |
| Hv--5H | Hv--transcript:HORVU.MOREX.r3.5HG0509430.1 | Zm--1 | Zm--transcript:Zm00001eb056760_T001 |
| Hv--5H | Hv--transcript:HORVU.MOREX.r3.5HG0512260.1 | Zm--1 | Zm--transcript:Zm00001eb058020_T001 |
| Hv--5H | Hv--transcript:HORVU.MOREX.r3.5HG0521030.1 | Zm--1 | Zm--transcript:Zm00001eb061300_T002 |
| Hv--5H | Hv--transcript:HORVU.MOREX.r3.5HG0524270.1 | Zm--1 | Zm--transcript:Zm00001eb062130_T001 |
| Hv--5H | Hv--transcript:HORVU.MOREX.r3.5HG0493040.1 | Zm--1 | Zm--transcript:Zm00001eb036040_T006 |
| Hv--5H | Hv--transcript:HORVU.MOREX.r3.5HG0495090.1 | Zm--1 | Zm--transcript:Zm00001eb036990_T003 |
| Hv--5H | Hv--transcript:HORVU.MOREX.r3.5HG0490730.1 | Zm--1 | Zm--transcript:Zm00001eb034940_T004 |
| Hv--5H | Hv--transcript:HORVU.MOREX.r3.5HG0486320.1 | Zm--1 | Zm--transcript:Zm00001eb037960_T001 |
| Hv--5H | Hv--transcript:HORVU.MOREX.r3.5HG0487550.1 | Zm--1 | Zm--transcript:Zm00001eb038320_T001 |
| Hv--5H | Hv--transcript:HORVU.MOREX.r3.5HG0484930.1 | Zm--1 | Zm--transcript:Zm00001eb041610_T001 |
| Hv--5H | Hv--transcript:HORVU.MOREX.r3.5HG0488000.1 | Zm--1 | Zm--transcript:Zm00001eb006680_T002 |
| Hv--5H | Hv--transcript:HORVU.MOREX.r3.5HG0430270.1 | Zm--1 | Zm--transcript:Zm00001eb031560_T003 |
| Hv--5H | Hv--transcript:HORVU.MOREX.r3.5HG0433720.1 | Zm--1 | Zm--transcript:Zm00001eb031010_T001 |
| Hv--5H | Hv--transcript:HORVU.MOREX.r3.5HG0515900.1 | Zm--1 | Zm--transcript:Zm00001eb059460_T002 |
| Hv--5H | Hv--transcript:HORVU.MOREX.r3.5HG0458030.1 | Zm--10 | Zm--transcript:Zm00001eb407240_T001 |
| Hv--5H | Hv--transcript:HORVU.MOREX.r3.5HG0487550.1 | Zm--2 | Zm--transcript:Zm00001eb101040_T001 |
| Hv--5H | Hv--transcript:HORVU.MOREX.r3.5HG0488000.1 | Zm--2 | Zm--transcript:Zm00001eb101190_T001 |
| Hv--5H | Hv--transcript:HORVU.MOREX.r3.5HG0488610.1 | Zm--2 | Zm--transcript:Zm00001eb101410_T006 |
| Hv--5H | Hv--transcript:HORVU.MOREX.r3.5HG0490730.1 | Zm--2 | Zm--transcript:Zm00001eb101880_T001 |
| Hv--5H | Hv--transcript:HORVU.MOREX.r3.5HG0493040.1 | Zm--2 | Zm--transcript:Zm00001eb102260_T001 |
| Hv--5H | Hv--transcript:HORVU.MOREX.r3.5HG0495090.1 | Zm--2 | Zm--transcript:Zm00001eb102580_T002 |
| Hv--5H | Hv--transcript:HORVU.MOREX.r3.5HG0484090.1 | Zm--2 | Zm--transcript:Zm00001eb099470_T002 |
| Hv--5H | Hv--transcript:HORVU.MOREX.r3.5HG0458030.1 | Zm--2 | Zm--transcript:Zm00001eb092180_T001 |
| Hv--5H | Hv--transcript:HORVU.MOREX.r3.5HG0486320.1 | Zm--2 | Zm--transcript:Zm00001eb100380_T001 |
| Hv--5H | Hv--transcript:HORVU.MOREX.r3.5HG0521030.1 | Zm--2 | Zm--transcript:Zm00001eb116410_T001 |
| Hv--5H | Hv--transcript:HORVU.MOREX.r3.5HG0458030.1 | Zm--3 | Zm--transcript:Zm00001eb139520_T002 |
| Hv--5H | Hv--transcript:HORVU.MOREX.r3.5HG0438460.1 | Zm--3 | Zm--transcript:Zm00001eb135040_T001 |
| Hv--5H | Hv--transcript:HORVU.MOREX.r3.5HG0486320.1 | Zm--4 | Zm--transcript:Zm00001eb176650_T001 |
| Hv--5H | Hv--transcript:HORVU.MOREX.r3.5HG0458030.1 | Zm--4 | Zm--transcript:Zm00001eb202130_T002 |
| Hv--5H | Hv--transcript:HORVU.MOREX.r3.5HG0512260.1 | Zm--5 | Zm--transcript:Zm00001eb214440_T001 |
| Hv--5H | Hv--transcript:HORVU.MOREX.r3.5HG0515900.1 | Zm--5 | Zm--transcript:Zm00001eb213550_T001 |
| Hv--5H | Hv--transcript:HORVU.MOREX.r3.5HG0519660.1 | Zm--5 | Zm--transcript:Zm00001eb212930_T001 |
| Hv--5H | Hv--transcript:HORVU.MOREX.r3.5HG0488610.1 | Zm--5 | Zm--transcript:Zm00001eb253350_T002 |
| Hv--5H | Hv--transcript:HORVU.MOREX.r3.5HG0486320.1 | Zm--7 | Zm--transcript:Zm00001eb314810_T001 |
| Hv--5H | Hv--transcript:HORVU.MOREX.r3.5HG0487550.1 | Zm--7 | Zm--transcript:Zm00001eb315330_T001 |
| Hv--5H | Hv--transcript:HORVU.MOREX.r3.5HG0488000.1 | Zm--7 | Zm--transcript:Zm00001eb315610_T002 |
| Hv--5H | Hv--transcript:HORVU.MOREX.r3.5HG0488610.1 | Zm--7 | Zm--transcript:Zm00001eb315910_T006 |
| Hv--5H | Hv--transcript:HORVU.MOREX.r3.5HG0490730.1 | Zm--7 | Zm--transcript:Zm00001eb316660_T002 |
| Hv--5H | Hv--transcript:HORVU.MOREX.r3.5HG0493040.1 | Zm--7 | Zm--transcript:Zm00001eb317460_T004 |
| Hv--5H | Hv--transcript:HORVU.MOREX.r3.5HG0495090.1 | Zm--7 | Zm--transcript:Zm00001eb318020_T003 |
| Hv--5H | Hv--transcript:HORVU.MOREX.r3.5HG0484090.1 | Zm--7 | Zm--transcript:Zm00001eb312850_T001 |
| Hv--6H | Hv--transcript:HORVU.MOREX.r3.6HG0605430.1 | Zm--10 | Zm--transcript:Zm00001eb428400_T001 |
| Hv--6H | Hv--transcript:HORVU.MOREX.r3.6HG0603090.1 | Zm--10 | Zm--transcript:Zm00001eb429090_T001 |
| Hv--6H | Hv--transcript:HORVU.MOREX.r3.6HG0605430.1 | Zm--2 | Zm--transcript:Zm00001eb072400_T001 |
| Hv--6H | Hv--transcript:HORVU.MOREX.r3.6HG0605430.1 | Zm--4 | Zm--transcript:Zm00001eb187720_T001 |
| Hv--6H | Hv--transcript:HORVU.MOREX.r3.6HG0607570.1 | Zm--4 | Zm--transcript:Zm00001eb188380_T001 |
| Hv--6H | Hv--transcript:HORVU.MOREX.r3.6HG0619980.1 | Zm--4 | Zm--transcript:Zm00001eb193920_T002 |
| Hv--6H | Hv--transcript:HORVU.MOREX.r3.6HG0624240.1 | Zm--4 | Zm--transcript:Zm00001eb192430_T001 |
| Hv--6H | Hv--transcript:HORVU.MOREX.r3.6HG0549910.1 | Zm--4 | Zm--transcript:Zm00001eb208200_T005 |
| Hv--6H | Hv--transcript:HORVU.MOREX.r3.6HG0545980.1 | Zm--4 | Zm--transcript:Zm00001eb209480_T001 |
| Hv--6H | Hv--transcript:HORVU.MOREX.r3.6HG0592420.1 | Zm--5 | Zm--transcript:Zm00001eb246520_T001 |
| Hv--6H | Hv--transcript:HORVU.MOREX.r3.6HG0603090.1 | Zm--5 | Zm--transcript:Zm00001eb250230_T002 |
| Hv--6H | Hv--transcript:HORVU.MOREX.r3.6HG0605430.1 | Zm--5 | Zm--transcript:Zm00001eb251200_T003 |
| Hv--6H | Hv--transcript:HORVU.MOREX.r3.6HG0606510.1 | Zm--5 | Zm--transcript:Zm00001eb251650_T003 |
| Hv--6H | Hv--transcript:HORVU.MOREX.r3.6HG0607570.1 | Zm--5 | Zm--transcript:Zm00001eb252100_T004 |
| Hv--6H | Hv--transcript:HORVU.MOREX.r3.6HG0608460.1 | Zm--5 | Zm--transcript:Zm00001eb252360_T001 |
| Hv--6H | Hv--transcript:HORVU.MOREX.r3.6HG0611310.1 | Zm--5 | Zm--transcript:Zm00001eb253350_T002 |
| Hv--6H | Hv--transcript:HORVU.MOREX.r3.6HG0624240.1 | Zm--5 | Zm--transcript:Zm00001eb257810_T001 |
| Hv--6H | Hv--transcript:HORVU.MOREX.r3.6HG0627040.1 | Zm--5 | Zm--transcript:Zm00001eb258240_T001 |
| Hv--6H | Hv--transcript:HORVU.MOREX.r3.6HG0545980.1 | Zm--5 | Zm--transcript:Zm00001eb229950_T002 |
| Hv--6H | Hv--transcript:HORVU.MOREX.r3.6HG0615060.1 | Zm--5 | Zm--transcript:Zm00001eb254900_T001 |
| Hv--6H | Hv--transcript:HORVU.MOREX.r3.6HG0580040.1 | Zm--5 | Zm--transcript:Zm00001eb240050_T001 |
| Hv--6H | Hv--transcript:HORVU.MOREX.r3.6HG0611310.1 | Zm--7 | Zm--transcript:Zm00001eb315910_T006 |
| Hv--6H | Hv--transcript:HORVU.MOREX.r3.6HG0624240.1 | Zm--9 | Zm--transcript:Zm00001eb374700_T001 |
| Hv--6H | Hv--transcript:HORVU.MOREX.r3.6HG0627040.1 | Zm--9 | Zm--transcript:Zm00001eb375660_T003 |
| Hv--6H | Hv--transcript:HORVU.MOREX.r3.6HG0615060.1 | Zm--9 | Zm--transcript:Zm00001eb372510_T001 |
| Hv--6H | Hv--transcript:HORVU.MOREX.r3.6HG0580040.1 | Zm--9 | Zm--transcript:Zm00001eb385110_T001 |
| Hv--7H | Hv--transcript:HORVU.MOREX.r3.7HG0679760.1 | Zm--1 | Zm--transcript:Zm00001eb034940_T004 |
| Hv--7H | Hv--transcript:HORVU.MOREX.r3.7HG0682660.1 | Zm--1 | Zm--transcript:Zm00001eb036040_T006 |
| Hv--7H | Hv--transcript:HORVU.MOREX.r3.7HG0684990.1 | Zm--1 | Zm--transcript:Zm00001eb036990_T003 |
| Hv--7H | Hv--transcript:HORVU.MOREX.r3.7HG0702010.1 | Zm--10 | Zm--transcript:Zm00001eb416800_T001 |
| Hv--7H | Hv--transcript:HORVU.MOREX.r3.7HG0682660.1 | Zm--2 | Zm--transcript:Zm00001eb102260_T001 |
| Hv--7H | Hv--transcript:HORVU.MOREX.r3.7HG0684990.1 | Zm--2 | Zm--transcript:Zm00001eb102580_T002 |
| Hv--7H | Hv--transcript:HORVU.MOREX.r3.7HG0679760.1 | Zm--2 | Zm--transcript:Zm00001eb101880_T001 |
| Hv--7H | Hv--transcript:HORVU.MOREX.r3.7HG0682660.1 | Zm--3 | Zm--transcript:Zm00001eb143520_T002 |
| Hv--7H | Hv--transcript:HORVU.MOREX.r3.7HG0702010.1 | Zm--4 | Zm--transcript:Zm00001eb173070_T001 |
| Hv--7H | Hv--transcript:HORVU.MOREX.r3.7HG0706460.1 | Zm--4 | Zm--transcript:Zm00001eb174220_T001 |
| Hv--7H | Hv--transcript:HORVU.MOREX.r3.7HG0684990.1 | Zm--4 | Zm--transcript:Zm00001eb175540_T003 |
| Hv--7H | Hv--transcript:HORVU.MOREX.r3.7HG0658960.1 | Zm--4 | Zm--transcript:Zm00001eb192430_T001 |
| Hv--7H | Hv--transcript:HORVU.MOREX.r3.7HG0708530.1 | Zm--4 | Zm--transcript:Zm00001eb204110_T001 |
| Hv--7H | Hv--transcript:HORVU.MOREX.r3.7HG0708530.1 | Zm--5 | Zm--transcript:Zm00001eb238140_T001 |
| Hv--7H | Hv--transcript:HORVU.MOREX.r3.7HG0675770.1 | Zm--5 | Zm--transcript:Zm00001eb252360_T001 |
| Hv--7H | Hv--transcript:HORVU.MOREX.r3.7HG0666110.1 | Zm--5 | Zm--transcript:Zm00001eb254900_T001 |
| Hv--7H | Hv--transcript:HORVU.MOREX.r3.7HG0716830.1 | Zm--5 | Zm--transcript:Zm00001eb240050_T001 |
| Hv--7H | Hv--transcript:HORVU.MOREX.r3.7HG0658960.1 | Zm--5 | Zm--transcript:Zm00001eb257810_T001 |
| Hv--7H | Hv--transcript:HORVU.MOREX.r3.7HG0660920.1 | Zm--6 | Zm--transcript:Zm00001eb278470_T003 |
| Hv--7H | Hv--transcript:HORVU.MOREX.r3.7HG0724770.1 | Zm--6 | Zm--transcript:Zm00001eb275820_T002 |
| Hv--7H | Hv--transcript:HORVU.MOREX.r3.7HG0706460.1 | Zm--6 | Zm--transcript:Zm00001eb260340_T001 |
| Hv--7H | Hv--transcript:HORVU.MOREX.r3.7HG0748480.1 | Zm--6 | Zm--transcript:Zm00001eb272150_T001 |
| Hv--7H | Hv--transcript:HORVU.MOREX.r3.7HG0682660.1 | Zm--7 | Zm--transcript:Zm00001eb317460_T004 |
| Hv--7H | Hv--transcript:HORVU.MOREX.r3.7HG0684990.1 | Zm--7 | Zm--transcript:Zm00001eb318020_T003 |
| Hv--7H | Hv--transcript:HORVU.MOREX.r3.7HG0658960.1 | Zm--7 | Zm--transcript:Zm00001eb312850_T001 |
| Hv--7H | Hv--transcript:HORVU.MOREX.r3.7HG0671700.1 | Zm--7 | Zm--transcript:Zm00001eb315870_T001 |
| Hv--7H | Hv--transcript:HORVU.MOREX.r3.7HG0682660.1 | Zm--8 | Zm--transcript:Zm00001eb364850_T001 |
| Hv--7H | Hv--transcript:HORVU.MOREX.r3.7HG0724770.1 | Zm--9 | Zm--transcript:Zm00001eb388300_T001 |
| Hv--7H | Hv--transcript:HORVU.MOREX.r3.7HG0708530.1 | Zm--9 | Zm--transcript:Zm00001eb383050_T001 |
| Hv--7H | Hv--transcript:HORVU.MOREX.r3.7HG0655660.1 | Zm--9 | Zm--transcript:Zm00001eb375660_T003 |
| Hv--7H | Hv--transcript:HORVU.MOREX.r3.7HG0658960.1 | Zm--9 | Zm--transcript:Zm00001eb374700_T001 |
| Hv--7H | Hv--transcript:HORVU.MOREX.r3.7HG0660920.1 | Zm--9 | Zm--transcript:Zm00001eb374120_T001 |
| Hv--7H | Hv--transcript:HORVU.MOREX.r3.7HG0666110.1 | Zm--9 | Zm--transcript:Zm00001eb372510_T001 |
| Hv--7H | Hv--transcript:HORVU.MOREX.r3.7HG0716830.1 | Zm--9 | Zm--transcript:Zm00001eb385110_T001 |
